# ING Proteins: Tumour Suppressors or Oncoproteins

**DOI:** 10.3390/cancers13092110

**Published:** 2021-04-27

**Authors:** Karine Jacquet, Olivier Binda

**Affiliations:** 1 Institut NeuroMyoGène (INMG), Université Claude Bernard Lyon 1, Université de Lyon, CNRS UMR 5310, INSERM U 1217, 69008 Lyon, France; karine.jacquet@univ-lyon1.fr; 2 Department of Cellular and Molecular Medicine, Faculty of Medicine, University of Ottawa, Ottawa, ON K1H 8M5, Canada

**Keywords:** oncoproteins, plant homeodomain, PHD, INhibitor of Growth, ING, histone mark reader, chromatin, cancer

## Abstract

**Simple Summary:**

The INhibitor of Growth (ING) family of proteins was founded when in 1996 ING1 was identified as a tumour suppressor, a protein that prevents cancer development. Important subsequent genetic and biochemical work in different models, showed that most ING family members are actually required for cellular proliferation, a hallmark of cancer cells. Although several studies suggest that INGs are broadly lost in cancer, some ING family members are amplified and correlate with a bad prognosis. This is especially true in hormone-dependent cancers, such as breast and prostate tumours. Herein, we review these studies and propose that ING proteins are not all tumour suppressors, but can play opposite role. Unquestionably, INGs have various functions, likely many yet to be discovered, and play complex roles during cancer development.

**Abstract:**

The INhibitor of Growth family was defined in the mid-1990s by the identification of a tumour suppressor, ING1, and subsequent expansion of the family based essentially on sequence similarities. However, later work and more recent investigations demonstrate that at least a few ING proteins are actually required for normal proliferation of eukaryotic cells, from yeast to human. ING proteins are also part of a larger family of chromatin-associated factors marked by a plant homeodomain (PHD), which mediates interactions with methylated lysine residues. Herein, we discuss the role of ING proteins and their various roles in chromatin signalling in the context of cancer development and progression.

## 1. Introduction

Eukaryotic genomes are compacted within the nucleus of the cell. Essentially, the genomic DNA is spooled around histone octamers composed of two copies of each histone—H2A, H2B, H3, and H4—forming the basic repeating unit of chromatin, the nucleosome. Given its role in compacting the genome, the nucleosome obstructs DNA transactions (i.e., transcription, recombination, replication, and repair). To regulate access to genetic information, the cell utilises various molecular mechanisms, including DNA methylation and histone modifications, to elicit access.

The chromodomain of the heterochromatin protein HP1α was identified in 2001 as an adaptor that potently associates with the histone H3 trimethylated on lysine 9 (H3K9^me3^) [1]. Since then, several other chromatin proteins demonstrated the capacity to distinguish and associate with various modifications on core and linker histones [2]. As such, these proteins are referred to as histone mark readers. These readers regulate access to the genetic information by associating with specific histone marks and stabilizing enzymatic activities to either open up or close the structure of the chromatin fibre.

**Figure 1:**
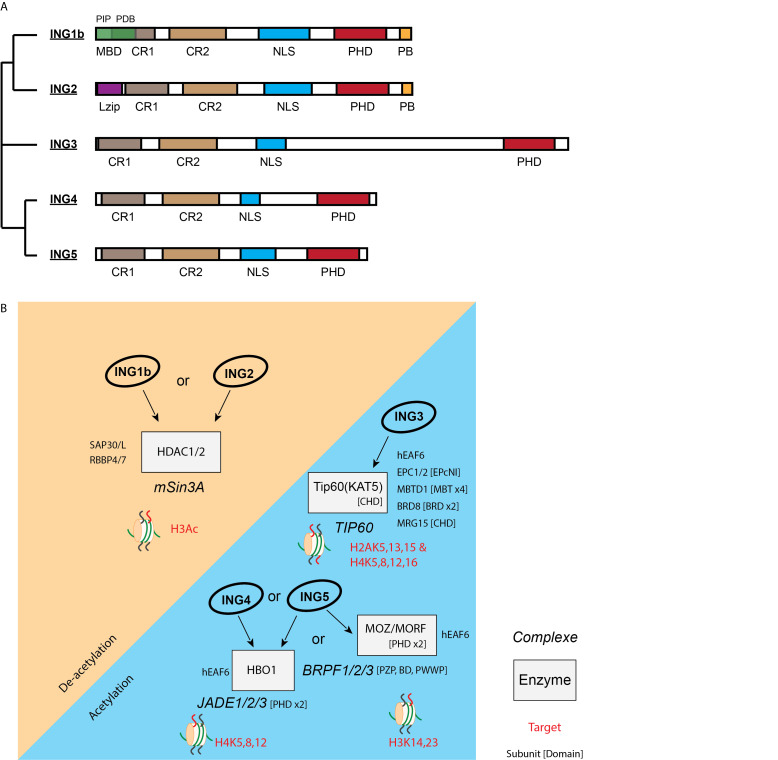
INhibitor of Growth (ING) proteins’ structure and complexes. (**A**) Phylogenic tree, domains, and conservation of the ING family. MBD: metal-binding domain responsible for zinc binding unique to ING1b. The MBD contains two subdomains: a PIP domain (PCNA-interacting protein motif) and a PDB domain (partial bromodomain); CR1 and 2: conserved regions 1 and 2; Lzip: leucine zipper-like region; NLS: nuclear localisation sequence; PHD: plant homeodomain responsible for binding to H3K4^me3^; PB: polybasic region unique to ING1 and ING2. ING4 and ING5 are paralogs that may form homo- or heterodimers in the cell [17,18]. ING1b and ING2 are close due to the presence of the PB motif in C-terminus. (**B**) ING protein association and targets. ING proteins are part of multiprotein complexes. There is a variable complexity of these complexes: from the TIP60 complex, which has around 18 stable subunits, to JADE or BRPF complexes, which harbour 5 subunits. INGs are all associated with an enzymatic activity implicated in the regulation of chromatin acetylation, on one side associated with the MYST (MOZ, Ybf2/Sas3, Sas2, and Tip60) family of acetyltransferases (HBO1 (KAT7), MOZ (KAT6A), MORF (KAT6B), Tip60 (KAT5) for ING3/4/5) but on the other side associated with the deacetylases HDAC1 (RPD3L1) and HDAC2 from the class I family of enzymes for ING1/2. The other subunits play an important targeting role in the ING complexes like the scaffolding subunits JADE (double PHD), BRBF (PZP, BD and PWWP), or EPC1/2 (EPcNI). Subunits having targeting function through chromatin reader domains are mentioned.

The INhibitor of Growth (ING) family is composed of five closely related paralogs (Figure 1A). They all associate with enzymatic activities via their amino terminus and bind methylated histones through a plant homeodomain (PHD) at the carboxy terminus [3]. Specifically, ING1 and ING2 associate with the histone deacetylase (HDAC) mSIN3A/HDAC1 complex [4,5], while ING3, ING4, and ING5 associate with TIP60, HBO1, and HBO1 or MOZ/MORF histone acetyltransferases (HAT), respectively [6,7] (Figure 1B). Initially defined as chromatin readers, INGs harbour a PHD domain that mediates protein–protein interaction with the histone H3 trimethylated on lysine 4 (H3K4^me3^), a histone mark commonly found at the transcriptional start site (TSS) of expressed genes (see [8] for review). By bridging HAT or HDAC activity to H3K4^me3^ at TSS, INGs can activate [9] or silence [10,11] gene expression, respectively, through gene accessibility/chromatin remodelling [12]. It is evident that ING proteins target their partners to specific regions of the genome through H3K4^me3^. However, they may also drive specific interactions with methylated nonhistone proteins such as p53, leading to their modification by either HAT or HDAC complexes [13,14]. Moreover, the PHD domains of ING1 and ING2 have also been described as binding important nuclear signalling molecules called phosphatidylinositol phosphates [15,16].

Over two decades ago, groundbreaking work identified ING1 as a tumour suppressor [19], and as such most studies of ING proteins have focused on their cancer-protective potential [20]. Unfortunately, almost all investigations merely correlate levels of ING proteins in various cancers without directly linking the functions of ING proteins with tumour suppression [21]. In addition, the evidence that ING proteins could be tumour suppressors is largely based on supraphysiological exogenous overexpression in cancer cell lines put under stress conditions (e.g., [22,23,24,25]). Investigations in transgenic mouse models have so far seemed to confirm that *Ing1* and *Ing2* are tumour suppressor genes, respectively preventing lymphomas and soft-tissue sarcomas [26,27,28]. However, recent investigations demonstrate that other ING proteins might actually be required for normal proliferation [29,30,31,32]. Thus, this short review will discuss ING proteins’ pleiotropic functions during oncogenesis.

## 2. Cellular Functions

**Figure 2:**
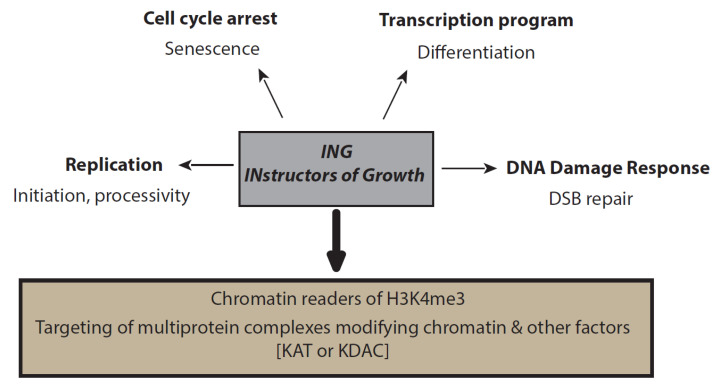
ING functions. ING proteins are peculiar chromatin readers with a PHD domain. They target their respective complexes to specific regions of the genome, where the different ING-containing complexes are responsible for posttranslational modifications (PTMs) of histones, as well as nonhistone proteins. These PTMs are involved in the regulation of many pathways during transcription, replication, DNA damage, and cell cycle arrest processes.

INGs’ functions can hardly be discriminated from their complex roles. Generally, defects for the different unique subunits show similar or related phenotypes. However, understanding ING-specific functions as part of the different complexes can be difficult [33,34]. Nonetheless, common functions seem to be shared among the ING family (Figure 2).

### 2.1. ING Proteins and DNA Damage

ING1 and ING2 function within the mSIN3A/HDAC1 complex [6]. This complex seems recruited to DNA damages to induce hypoacetyled H3K56 and the nonhomologous end joining (NHEJ) pathway [35,36]. Acetylation of chromatin is observed to open the damage sites and grant access to the repair machinery [37]. Thus, mSIN3A/HDAC1 might also be engaged in restoring the chromatin landscape after DNA repair [38,39]. Functionally, the PHD domain of ING2 targets the mSIN3A/HDAC1 complex to cell cycle promoters (e.g., *CCND1* gene encoding cyclin D1) in response to DNA damages [10]. An intact aromatic cage is also required for ING1 to facilitate DNA repair after UV irradiation and induce cell death [40]. In parallel, ING1 PIP domain (PCNA interacting protein domain) binds PCNA in response to UV radiation [41] (Figure 1A). PCNA acetylation should be tightly regulated, as it is interrelated with other critical posttranslational modifications (PTM), potentially affecting its structure and function during genome replication and repair [42].

Notably, ING3 within the TIP60 complex regulates DNA double-strand break (DSB) repair and influences the choice between NHEJ and homologous recombination (HR) repair pathways [43]. Moreover, ING3 was recently found to directly regulate ATM signalling at DSBs [44], as previously shown for Tip60 [45]. Other ING homologs were found to play a role during the DNA damage response in yeast and nematode [46,47]. Similarly, in response to DNA damages, ING4 appears to associate with H3K4^me3^ at gene promoters (i.e., *SMC4*) to regulate their expression and induce apoptosis [9]. Finally, ING5 also seems to play a role during the DNA damage response (DDR). Specifically, an increased level of ING5 is observed upon damages, followed by its translocation into the nucleus [48].

### 2.2. Cell Cycle, Cellular Senescence, and Apoptosis

In a simplified view, tumour suppression can function by inducing either apoptosis or a permanent state of exit from the cell cycle called senescence [49]. Interestingly, senescent cells have a marked increase in ING1 levels while silencing of ING1 increases proliferative lifespan [50]. Similarly, ING2 levels are elevated in late-passage human fibroblasts and its overexpression induces senescence, which could be delayed by ING2 silencing [51]. Interestingly, inhibition of the ING5-associated MOZ/MORF acetyltransferases also induces senescence and prevents tumour growth [52].

ING proteins are well-known regulators of the p53 pathway [13,14,25]. They can act at different levels, mostly transcriptionally, but also through direct modification of p53 by acetylation/deacetylation inducing the regulation of the G_1_/S transition. For example, ING3, as part of TIP60, upregulates the p53 pathway through p21 transcription and induces cell cycle arrest [53,54,55]. The ING3-containing complex TIP60 is also responsible for p53 acetylation leading to the activation of apoptosis [53,56,57]. Overexpression of ING3 in colon cancer cell lines induces apoptosis and inhibits tumour growth [25], whereas downregulation of ING3 is associated with a poor prognosis in cutaneous melanoma [58].

Of note, ING4 expression in normal fibroblasts induces proliferation arrest and a senescence-associated secretory phenotype (SASP) [59]. In agreement with previous work showing that SASP stimulates tumour progression in the tissue microenvironment [60], these ING4-induced senescent cells promote tumour growth in mice [59].

Finally, ING5 can interact with the replication machinery. The HBO1 (histone acetyltransferase binding to ORC1) complex is recruited at replication origins for hyperacetylation of H3 [61,62]. Similarly, ING5 has been described as interacting with the minichromosome maintenance helicases (MCM) complex and is critical for normal progression through the S phase [6].

### 2.3. Other Functions of the INGs

ING4 interacts with the NFκB pathway to suppress angiogenesis in glioma, colorectal, and breast cancers [63,64,65]. ING4 also represses HIF (hypoxia-inducible factors) and regulates its transcriptional activity as well [66]. Hence, ING4 plays a key antitumorigenic function. ING1 seems to play a similar role through the regulation of angiopoietins [67].

## 3. ING Family Roles in Cancer

### Expression of ING Proteins in Cancer

The levels of ING genes and proteins have been reported to be lost in several studies involving a restricted number of cases (reviewed in [3,19]). However, contrarily to what would be expected from a family of genes called INhibitor of Growth, ING genes are, according to cBioPortal for Cancer Genomics [68,69], broadly altered, i.e., amplified, deleted, or mutated in cancers, but at moderate levels (usually below a frequency of 10%).

Although previous investigations have reported a loss of ING3 expression in small sample number studies, we found, using the cBioPortal, that INGs are broadly amplified in a variety of cancers [32]. Using a high-throughput tissue microarray approach consisting of benign prostate hyperplasia and prostate cancer samples, we have validated that ING3 protein levels are elevated in prostate cancer, corresponding to cBioPortal genomic data [32]. Moreover, although altered in non-small-cell lung cancers, ING2 seems to be required for cancer proliferation [70]. In other words, the expression of ING proteins in cancer does not always correlate with cell proliferation.

## 4. ING Proteins in Eukaryotic Model Organisms

### 4.1. Yeast Deletion Models

ING proteins are conserved in unicellular eukaryotes, such as *Saccharomyces cerevisiae*, which has three ING-like proteins called Yng1, Yng2, and Pho23 [46]. The closest relatives of Yng1 are ING4/5, while Yng2 is related to human ING3. Since Pho23 is associated with Rpd3 [71], it would be related to ING1 and ING2. Yng2 associates with Tra1 (TRRAP in mammals) [46] through a direct interaction with Epl1, further supporting that Yng2 and ING3 are related as ING3 associates with the TIP60/TRRAP complex [6]. Notably, the three yeast ING homologs (i.e., Pho23, Yng1, and Yng2) were purified as stable components of Sin3/Rpd3 deacetylase complex or NuA3 and NuA4 acetyltransferase complexes, respectively [72,73,74].

Counterintuitively, ΔYng2 *S. cerevisiae* cells grow slower than wild-type cells (~5 h doubling time versus ~3 h normally) and are multibudded, while ΔYng1 and ΔPho23 grow normally, but combined ΔYng1/ΔYng2/ΔPho23 deletion leads to further growth impairment (~10 h doubling time) [46]. Moreover, both ΔYng1 and ΔYng2 cells fail to grow from nonfermentable carbon sources (e.g., galactose, glycerol) [46]. Mild growth defects were revealed in the ΔPho23 strain grown on galactose or glycerol, but also in response to heat stress [46]. Importantly, the expression of human ING1 or *Schizosaccharomyces pombe* Png1 can rescue the ΔYng2 growth defect phenotypes [46]. These experiments demonstrate that the functions of ING proteins are conserved between unicellular eukaryotes and humans but also highlight that, contrary to what their names suggest, at least some ING family members are actually required for growth [74].

A number of potential mechanisms have been suggested for the growth defects observed in yeast models. The Yng2 deletion impairs progression through mitosis and meiosis [75] but is also required to overcome DNA damage-induced S phase arrest [76]. As in humans, Yng2 (ING3) and Yaf9 (YEATS4/GAS41) associate with the acetyltransferase Esa1 (Tip60/KAT5). Interestingly, both ΔYaf9 and ΔYng2 have growth defects that are enhanced by spindle stress [77], supporting their role during chromosome segregation in M phase.

In addition, the ΔPho23 strain displays elevated levels of autophagy gene expression, along with corresponding ATG proteins, as well as increased autophagy activity upon nitrogen starvation [78].

Finally, genetic deletion of the *S. pombe* ING ortholog Png1 also results in slower proliferation [46,79].

### 4.2. Ing1^−/−^ Mouse Model

Interestingly, deletion of all *Ing1* isoforms in CJ-7 mouse strain predisposes animals to lymphomas [27], suggesting that Ing1 is indeed a tumour suppressor. Moreover, disruption of the p37Ing1 isoform in 129/SVJ strain, while leaving the p31Ing1 isoform intact, was sufficient to increase the incidence of follicular B-cell lymphomas [26]. Since p31Ing1 remains intact in the p37Ing1-null animals, this suggests that p31Ing1 does not compensate for the loss of p37Ing1 even though the levels of p31Ing1 are elevated in p37Ing1-null tissues [26].

Although CJ-7 *Ing1^−/−^* mouse embryonic fibroblasts (MEF) have a normal replicative lifespan and appear to have normal response to growth-inhibitory treatments, they display mild cell cycle profile alterations in response to various genotoxic insults with a reduced G_2_ population [27]. Comparably, 129/SVJ p37Ing1-null MEFs had increased growth rates [26]. Interestingly, the reduced body weight reported for CJ-7 *Ing1*^−/−^ animals is in agreement with growth defects observed in the ΔYng2 *S. cerevisiae* strain.

Interestingly, knockout of all Ing1 isoforms in TBV-2 strain results in MEFs that are resistant to RasV12-induced senescence [80]. Oncogene-induced senescence (OIS) is triggered by increased Ras/Raf/MAPK signalling through mutations (e.g., RasV12), upstream cascade activation (e.g., EGFR), or downstream loss of pRB function and increased E2F1 activity. It remains unknown how loss of Ing1 prevents RASV12-induced senescence and if or how and where it regulates the signalling cascade. Since Ing1 is required for OIS, it is likely acting downstream of Ras.

### 4.3. Ing2^−/−^ Mouse Model

Ing2 mRNA is highly expressed in testes, followed by lung and spleen [28]. In agreement with a role in testes biology, male *Ing2*^−/−^ 129/SV mice had smaller testes, lower sperm counts, and impaired sperm motility, with abnormal morphology, which resulted in male infertility [28].

Although the authors did not demonstrate that Ing2 regulates directly the genes that they have identified by microarray, they have found that a number of sperm (e.g., *Prss21*, *Sly*, *Spef2*) and chromatin (e.g., *Setdb2*, *Suv39h2*, *Ing3*) genes were downregulated in *Ing2*^−/−^ testes [28]. Since ING3 is required for cellular proliferation [32], decreased Ing3 levels in *Ing2*^−/−^ testes may hypothetically explain smaller testes in *Ing2*^−/−^ males. In addition, p53 and p53-regulated apoptotic factor PUMA were upregulated in *Ing2*^−/−^ testes, which correlated with increased apoptotic cells, while senescence appeared unaffected [28].

Importantly, *Ing2*^−/−^ mice were afflicted by increased soft-tissue sarcoma [28], demonstrating that loss of Ing2 is sufficient to increase tumorigenicity and that Ing2 is likely a bona fide tumour suppressor.

### 4.4. Ing3^−/−^ Mouse Model

So far, there is one recent report of *Ing3* KO mice [81]. In this study, as expected, *Ing3*^−/−^ mice were embryonic lethal at stage E10.5. Thus, they display very strong growth defects as well as severe developmental disorders of the nervous system, revealing a lack of ectodermal differentiation. Tip60 KO causes early embryonic lethality near blastocyst stage and embryo death before implantation [82,83]. However, because of the embryonic lethal phenotype, it could not be concluded that ING3 functions as an oncoprotein or a tumour suppressor.

### 4.5. Ing4^−/−^ Mouse Model

Lipopolysaccharides (LPSs) are membrane components of Gram-negative bacteria and trigger a potent immune-inflammatory response by activating the NFκB transcription factor. The ING protein ING4 was found to regulate NFκB-dependent transcription and thus is hypothesised to be involved in inflammatory responses. Basically, LPS binds to the toll-like TLR4 receptor, activating a signalling cascade that results in the phosphorylation of the NFκB inhibitor IκB by IKK1/2, phospho-dependent degradation of IκB, and expression of NFκB-responsive genes [84].

Interestingly, the *Ing4*^−/−^ mouse model is viable; the mice developed normally and remained free of tumours over the 20-month period investigated [85]. However, *Ing4*^−/−^ mice expressed high levels of inflammatory cytokines (e.g., IL6) and died within 24 h of exposure to LPS.

In agreement with the *Ing4*^−/−^ model, small interfering RNA-mediated silencing of ING4 prevents normal cell cycle progression of human breast cancer MCF7 cells, marked with an accumulation in the G_2_/M phase of the cell cycle [6]. Together, these results suggest that, although ING4 is involved to some degree in the regulation of the cell cycle, its principal function is not to suppress tumour development.

### 4.6. Ing5^−/−^ Mouse Model

To our knowledge there are no published *Ing5*^−/−^ knockout mouse models yet, thus limiting the interpretation of ING5′s role as a candidate tumour suppressor.

### 4.7. Cell-Based Gene Silencing

Silencing of either HBO1 (ING4 complex subunit) or TIP60 (ING3 complex subunit) acetyltransferases by shRNA reduced proliferation of human embryonic kidney cells (HEK293T), which accumulated in G_2_/M [6], a phenotype also observed in yeast [7]. In agreement, knockout of ING4 in myelogenous leukaemia HAP1 cells led to reduced proliferation [86]. Silencing of HBO1 in human breast cancer cells (MCF7) also decreased BrdU incorporation, suggesting a role in DNA replication [6]. In contrast, silencing of ING4 only modestly reduced BrdU incorporation in MCF7 cells but led to an increase in G_2_/M population. However, silencing of ING5, which also associates with HBO1, led to the disappearance of the G_2_/M phase population and entirely blocked DNA synthesis [6]. Interestingly, ING5-silenced human osteosarcoma cells (U2OS) are partially resistant to DNA damage-induced apoptosis [48]. Moreover, two independent shRNA targeting ING5 inhibited the growth of HCT116, HCT116-p53^−/−^, U2OS, and HeLa cell lines, possibly by inducing apoptosis [87]. In agreement, silencing of ING5 in primary human keratinocytes reduced clone formation in an organotypic skin reconstitution assay due to terminal differentiation [88].

In agreement with the requirement of TIP60 for cellular proliferation [6], we observed that ING3 was also essential for the proliferation of human breast (MCF7) and prostate (LNCaP, PC3) cancer cell lines [32], suggesting a central role for the ING3/TIP60 complex in cell cycle progression. Indeed, ING3 regulates the expression of cell cycle genes by interacting with H3K4^me3^ at transcriptional start sites [32]. In addition, silencing of ING3 induces cellular differentiation and promotes adipogenesis using a human mesenchymal progenitor cell-derived osteoblast cellular model [44], again highlighting the requirement of ING3 for sustained cellular proliferation in at least these instances. Interestingly, cell-based silencing experiments recently demonstrated that ING3 is necessary for proper nonhomologous end joining (NHEJ) and homologous recombination (HR) DNA damage repair pathways [44], potentially leading to cell cycle arrest, in agreement with previous work [32,46].

### 4.8. Other Models

INGs have been studied in other models. In *Caenorhabditis elegans*, ING3 activates apoptosis induced by DNA damages through a p53-dependent pathway [47]. In *Xenopus laevis*, several splice variants were identified for ING1 (seven variants so far), all playing a positive role in apoptosis [89].

## 5. Reclassification of ING Proteins

There is increasing evidence that ING proteins have different, sometimes opposite, functions. Animal models clearly define ING1 and ING2 as bona fide tumour suppressors. However, yeast models suggested early on that ING proteins are required for cellular proliferation under some environmental conditions. In support of this hypothesis, we have recently found that ING3 (which is, based on sequence similarity, the closest relative to yeast Yng2, an ING member required for growth in a nonfermentable carbon-source environment) is also required for cellular proliferation of human breast and prostate cancer cells [32].

In agreement with yeast models, we found that ING3 is required for proliferation of breast, ovarian, and prostate cancer cells [32], suggesting that it may function as an oncoprotein instead of a tumour suppressor. Indeed, increased ING3 levels in prostate cancer patient samples correlate with poor survival. Moreover, ING3 expression in normal human cells was sufficient for transformation [32].

Thus, ING proteins should more accurately be called INstructors of Growth, to retain the gene symbols and refer to cell cycle regulators instead of candidate tumour suppressors, to better reflect the broad pleiotropic functions of the family members.

## 6. Conclusions

In light of limited previous observations and recent systematic approaches, it appears that ING3 is not a tumour suppressor, at least in some cancers [29,32]. Interestingly, ING4 and ING5 are also required for proliferation of breast cancer cells, supporting the hypothesis that ING proteins play pleiotropic functions in eukaryotic cells.

Alternative splicing might be a mechanism that modulates ING proteins’ functions, as for ING1 [90,91]. Most probably, posttranslational modifications (PTMs) of the ING proteins should be considered as a way to switch/modulate their functions ON and OFF. Among those modifications, acetylation is central because we already know that HAT complexes can autoacetylate, not only on the catalytic subunit but also on the other subunits [92]. Furthermore, PTM of the PHD in particular is already documented for ING4 and ING5 [93]. Those PTMs could regulate their half-life (i.e., ubiquitylation), subcellular localization (i.e., PTM in NLS), and interaction with partner subunits or interactors (through phosphorylation and others) (for review, see [94]). For example, ING4 was reported to be phosphorylated on threonine 197 (T197) and tyrosine 198 (Y198) [95]. The latter is one of the residues composing the H3K4^me3^-binding aromatic cage [9]. Based on mutational analyses of other readers, phosphorylation of ING4-Y198 would likely impact its interaction with H3K4^me3^ [96].

We thus propose to rename the ING proteins the INstructors of Growth family of histone mark readers to retain the gene symbol by which they are known, while highlighting the inhibiting and stimulating impact on proliferation of ING1–2 and ING3–5, respectively. Much work remains to definitively characterise the expanding cellular roles of the ING family of epigenetic regulators in normal and pathological states.
